# Effect of dispase denudation on amniotic membrane

**Published:** 2009-09-25

**Authors:** Laurence S. Lim, Andri Riau, Rebekah Poh, D.T. Tan, R.W. Beuerman, J.S. Mehta

**Affiliations:** 1 Singapore National Eye Center, Singapore; 2 Singapore Eye Research Institute, Singapore; 3 Department of Ophthalmology, Yong Loo Lin School of Medicine, National University of Singapore, Singapore

## Abstract

**Purpose:**

To describe the cellular components, biochemical composition, and membrane surface characteristics of denuded human amniotic membrane (DHAM) treated with Dispase II.

**Methods:**

DHAM was incubated with Dispase II (1.2 U/ml) for 30 min, 60 min, or 120 min. This was followed by gentle scraping to remove any remaining epithelial cells using a cell scraper. Histology, immunohistochemistry for extracellular matrix molecules and growth factors, and transmission (TEM) and scanning electron microscopy (SEM) were performed to assess the effects of increasing durations of incubation on DHAM structure.

**Results:**

Dispase II treatment was associated with the digestion of several ECM molecules, particularly those in the basement membrane including collagen VI, fibronectin, and laminin. FGF-2 and PDGF-B expression were unaffected by Dispase II, but TGF-α, TGF-β1, TGF-β2R, PDGF-A, VEGF, and EGFR expression were all reduced by Dispase II incubation. TEM confirmed the disruption of DHAM ultrastructure with increasing duration of Dispase II incubation, beginning with disruption of the basal lamina and progressing to loosening of the stromal collagen network as well.

**Conclusions:**

The use of Dispase II in the preparation of DHAM causes significant changes to the ultrastructure of the membrane, particularly the BM. Prolonged incubation with dispase may cause significant disruption in DHAM structure which may affect cell growth in cultured explants.

## Introduction

Human amniotic membrane (HAM) has been used successfully in ocular surface reconstruction as a biological bandage and as a substrate for corneal, conjunctival, and limbal stem cell expansion [1-6]. Ultrastructurally, intact HAM is composed of a single layer of cuboidal epithelial cells attached to a basement membrane (BM) overlying a loose mesenchymal stroma [7]. The combination of anti-inflammatory, anti-microbial and antiviral, anti-fibrotic, and anti-angiogenic properties [8-13] of HAM provides a favorable environment for cellular attachment and expansion, with successful application in both in vivo and in vitro settings.

HAM is harvested by blunt dissection off the underlying chorion of a donor placenta following cesarean section. Numerous protocols have been described for further processing of fresh tissue, and there is currently no consensus on the best method of tissue preparation [14]. HAM has been used either with the epithelial cells intact but nonviable following cryopreservation, or with the membrane denuded of all epithelial cells. Neither preparation has been conclusively shown to be superior, but the general opinion [14-18] is that HAM with intact amniotic epithelial cells retards migration and differentiation of cultivated cells. In this regard, denudation of HAM may be performed either chemically with reagents e.g. EDTA [19], or enzymatically with proteolytic enzymes like dispase [20,21], or a combination trypsin/EDTA [22].

Dispase II is a neutral protease, crystallized from cultured *Bacillus polymyxa* [23], that has been shown to have high specificity for fibronectin and collagen IV, making it particularly suitable for cleaving epithelial-mesenchymal adhesions. Following the original description of its use to remove epidermis from skin [24], intact sheets of conjunctival, corneal, and limbal epithelium have also been successfully isolated following incubation with Dispase II [20,25,26]. There is currently little data on the effects of different Dispase II treatment regimes on the ultrastructure and biological properties of HAM [18,21,27,28].

The aim of the study was to describe the effects of Dispase II treatment and its duration on the cellular components, biochemical composition, and membrane surface characteristics of denuded HAM (DHAM).

## Methods

All experimental procedures conformed to the ARVO Statement for the Use of Animals in Ophthalmic and Vision Research and the guidelines of the Declaration of Helsinki for biomedical research involving human subjects and were approved by the Singapore National Eye Center and Singapore Eye Research Institute Ethics Committees.

### Procurement and preparation of human amniotic membrane

Human placentas were obtained from mothers who had undergone cesarean sections. The membranes were washed with phosphate buffered saline (PBS) to remove the blood clots. The HAM was peeled away from the chorion and flattened onto a sterilized nitrocellulose filter paper (Millipore, Bedford, MA). The HAM was then stored in 50% DMEM, 50% glycerol (Invitrogen-Gibco, Carlsbad, CA) at -80 ºC. In preparation for its use, the HAM was thawed, rinsed with PBS, and incubated with Dispase II (1.2 U/ml; Invitrogen-Gibco) for 30 min (30DHAM), 60 min (60DHAM) or 2 h (120DHAM). This was followed by gentle scraping to remove any remaining epithelial cells using a cell scraper (Greiner Bio-One GmbH, Frickenhausen, Germany.)

### Immunohistochemistry for extracellular matrix molecules and growth factors

Tissues were embedded in optimal cutting temperature freezing compound (OCT) (Tissue-Tek; Sakura Finetek, Torrance, CA). Sections (6 μm thick) were cut and positioned on poly-lysine coated glass slides and then air dried for 20 min. They were then subjected to Hematoxylin and eosin (H&E) staining or indirect-immunostaining analysis. The tissues were fixed in -20 °C acetone for 15 min, followed by washing three times for five min each time in PBS Triton X-100 (0.15%). The samples were then immersed for 1 h in blocking solution (1% BSA in 0.01M PBS) at room temperature in a humid chamber. After washing for five min in PBS Triton-0.15% they were incubated overnight, at 4 ºC, with primary antibody solutions (Table 1). Each sample (30DHAM, 60DHAM, and 120DHAM) was tested in quadruplicate. Epithelialized, cryo-preserved HAM sections and normal mouse/goat/rabbit immunoglobulin were used as positive and negative controls, respectively. The tissues were then washed three times for five min each time in PBS (0.01 M), and were subsequently incubated at room temperature with fluorescein (FITC conjugated) secondary antibody (Alexa-488 labeled anti-mouse IgG, anti-rabbit IgG, anti-goat IgG; Chemicon International, Temecula, CA) at a dilution of 1:2,000. After 1 h, the tissues were washed 3 times for 5 min each time in PBS (0.0 1M), in the dark. Slides were then mounted with Fluorsave with DAPI (4,6-diamidino-2-phenylindole; Vector Laboratories, Burlingame, CA). They were subsequently examined with a Zeiss Axioplan 2 fluorescence microscope (Zeiss, Oberkochen, Germany).

**Table 1:** Antibodies used in the study.

**Antigen**	**Dilution IHC**	**Dilution WB**	**Type of Antibody**	**Immunized Animal**	**Company**
**Extracellular matrix**					
Fibronectin	1:100		Mo	M	Chemicon
Thrombospondin	1:20		Po	G	Santa Cruz
Elastin	1:20		Po	G	Santa Cruz
Tenascin	1:20		Mo	M	Zymed
Laminin 5	1:50		Mo	M	DAKO
Collagen I	1:10		Mo	M	Acris
Collagen II	1:50		Mo	M	Neomarkers
Collagen IV	1:50		Mo	M	Neomarkers
Collagen VI	1:100	1:200	Mo	M	Santa Cruz
Collagen VII	1:100		Mo	M	Neomarkers
**Growth factors**					
KGF	1:100	1:500	Po	R	Acris
TGFβ1	1:100	1:500	Mo	M	Acris
EGFR	1:100		Mo	M	Chemicon
TGFα	1:50	1:200	Po	R	Santa Cruz
TGF β2 recep	1:100		Po	R	Neomarkers
FGF	1:100	1:200	Mo	M	Upstate
VEGF	1:100		Mo	M	Santa Cruz
PDGF-A	1:200		Mo	M	Santa Cruz
PDGF-B	1:50	1:200	Mo	R	Neomarkers
IGF-1			Mo	M	Upstate

Abbreviations and companies are: Mo, monoclonal; Po, polyclonal; M, mouse; G, goat; R, rabbit Santa Cruz: Santa Cruz Biotechnology Inc., Santa Cruz, CA; Chemicon: CHEMICON International Inc., Temecula, CA; Acris: Acris GmbH, Germany; Neomarkers: Neomarkers Inc., Fremont, CA; Upstate: Upstate, Temecula, CA; DAKO: DAKO, Carpinteria, CA; Zymed: Zymed Laboratories, San Francisco, CA.

### Electron microscopy

#### Transmission electron microscopy (TEM)

DHAM tissues were fixed with cold 2% paraformaldehyde and 2% glutaraldehyde in 0.1M sodium cacodylate buffer, pH 7.4 (Electron Microscopy Sciences, Hatfield, PA) at 4 ^°^C overnight. Tissues were washed in sodium cacodylate buffer, rinsed with distilled water and trimmed into smaller pieces. Tissues were then post-fixed in 1% osmium tetroxide and potassium ferrocyanide (Electron Microscopy Sciences) to enhance membrane contrast. After extensive rinsing with distilled water, tissues were dehydrated in a graded series of ethanol, and embedded in Araldite (Electron Microscopy Sciences).

All semi-thin sections of 0.5-1 µm thickness were cut with Reichert-Jung Ultracut E Ultramicrotome (C. Reichert Optische Werke AG, Vienna, Austria), counter-stained with toluidine blue/basic fuchsin stain and examined using a Zeiss Light Microscope (Carl Zeiss, Germany). All ultra-thin sections of 60-80 nm were collected on copper grids, doubled-stained with uranyl acetate and lead citrate for 8 min each, then viewed and photographed on a JEM 1220 electron microscope (JEOL, Tyoko, Japan) at 100 kv.

#### Scanning electron microscopy (SEM)

Following denudation with Dispase at different time points, the treated HAMs were examined by electron microscopy. Specimens were immersed in a fixative containing 2.0% glutaraldehyde, 2% paraformaldehyde, and 0.1M sodium cocodylate (PH 7.4) overnight at 4 ºC. They were then transferred and stored in sodium cocodylate buffer (EMS, Hatfield, PA). Before processing, the samples were washed twice in distilled water for 10 min. They were then immersed in 1% osmium tetra-oxide (FMB, Singapore) for 2 h at room temperature. Following this, they were dehydrated in a graded ethanol series of 25%, 50%, 75%, 95% and 100% each for 10 min, the 100% being performed three times. The samples were then dried in a critical point dryer (BALTEC, Balzers, Liechtenstein) and mounted on SEM stubs using carbon adhesive tabs. They were then sputter coated with a 10 nm thick layer of gold (BALTEC) and examined with a scanning electron microscope (JSM-5600;JEOL, Tokyo, Japan) at 15 W.

## Results

### Histology

Histological examination of DHAM specimens incubated with Dispase II showed progressive disruption of the membrane with increasing durations of treatment.

**Figure 1:**
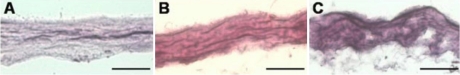
Hematoxylin-eosin (H&E) staining of denuded human amniotic membrane after treatment with Dispase II (1.2 U/ml) for 30 min (**A**), 60 min (**B**), and 120 min (**C**). The scale bars indicate 50 mm.

After 30 min, the DHAM retained a fairly compact structure with minimal surface convolutions and protrusions. After 120 min, the DHAM assumed a loose, disorganized structure with marked full thickness convolutions and surface protrusions (Figure 1).

### Immunohistochemistry of extracellular matrix molecules and growth factors

**Figure 2:**
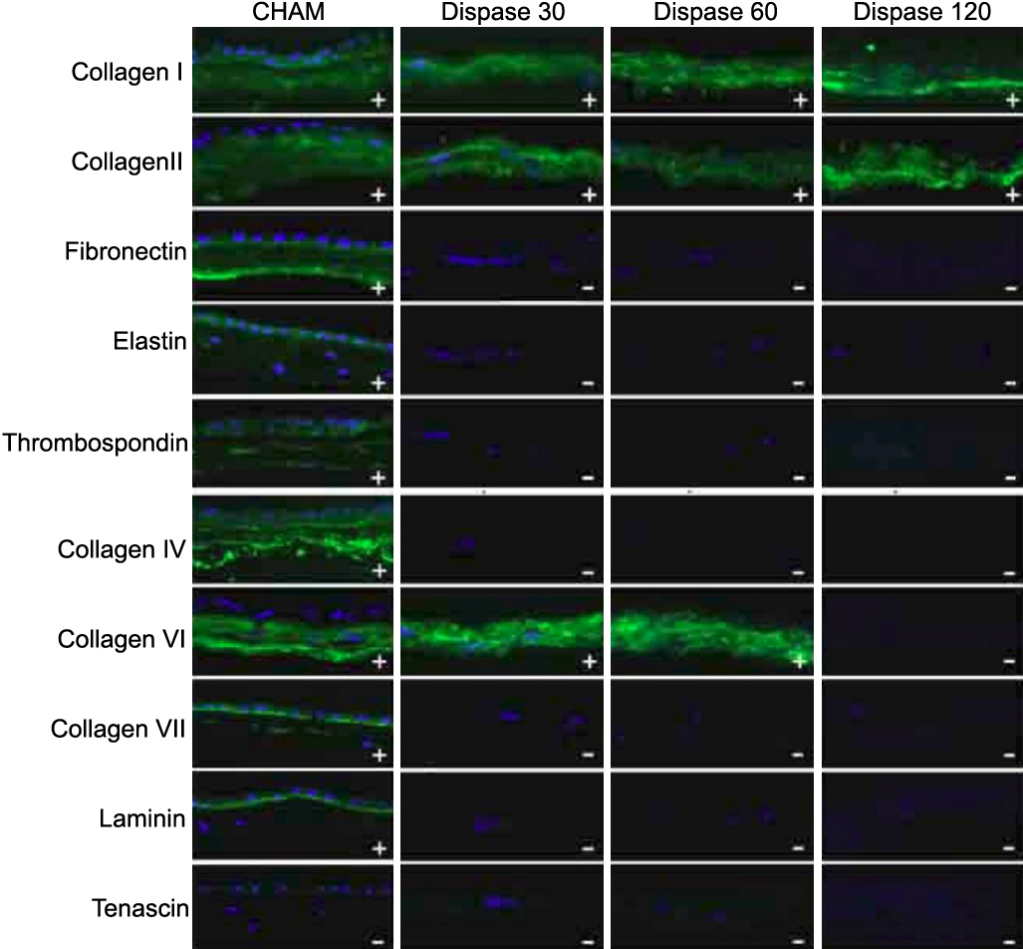
Expression of various extracellular matrix components in positive control, which is the intact cryo-preserved human amniotic membrane (CHAM) and denuded HAM after treatment with Dispase II (1.2 U/ml) for 30 min (Dispase30), 60 min (Dispase60), and 120 min (Dispase120). The “+” or “–“ signs in the bottom left of each image indicate the presence or absence of protein, respectively. The scale bar indicates 50 mm.

**Figure 3:**
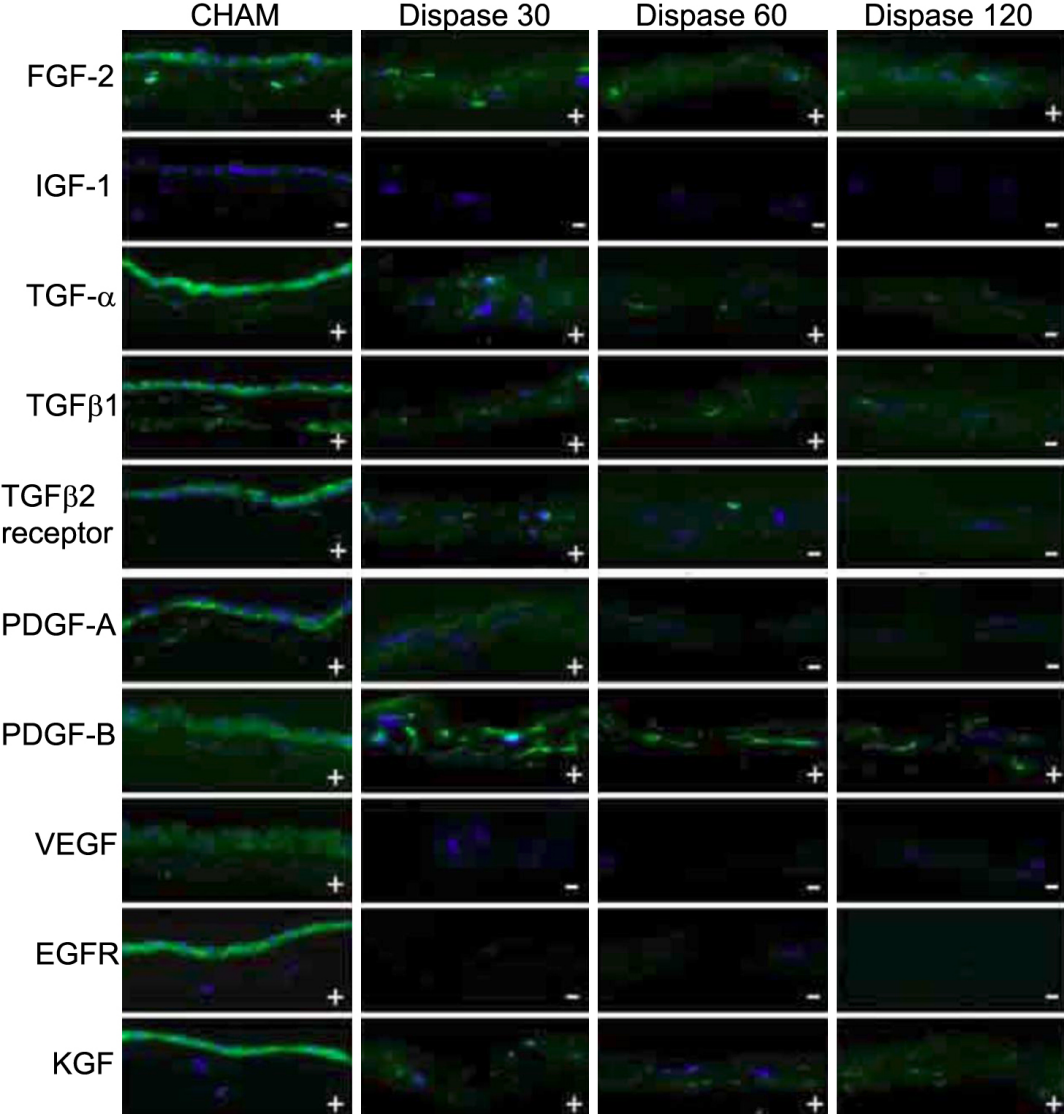
Expression of various growth factors (GFs) and GF receptors in the positive control, which is the intact cryo-preserved human amniotic membrane (CHAM) and denuded HAM after treatment with Dispase II (1.2 U/ml) for 30 min (Dispase30), 60 min (Dispase60), and 120 min (Dispase120). The “+” or “–“ signs in the bottom left of each image indicate the presence or absence of protein, respectively. The scale bar indicates 50 mm.

Sections incubated with mouse, goat, and rabbit immunoglobulins and without primary antibody demonstrated no immunoreactivity (data not shown). Compared to cryo-preserved human amniotic membrane (CHAM), DHAM expression of collagen I and II were comparable for all incubation periods, and for 30 and 60 min only for collagen VI (Figure 2). Collagen IV, VII, thrombospondin, elastin, fibronectin, tenascin, and laminin were not detectable in any DHAM samples. Amongst the growth factors of interest, FGF-2 and PDGF-B expression were comparable to CHAM for all time periods, reduced with increasing duration of incubation for TGF-α, TGF-β1, TGF-β2R, and PDGF-A and absent for VEGF and EGFR. (Figure 3)

### Transmission electron microscopy (TEM) and scanning electron microscopy (SEM)

**Figure 4:**
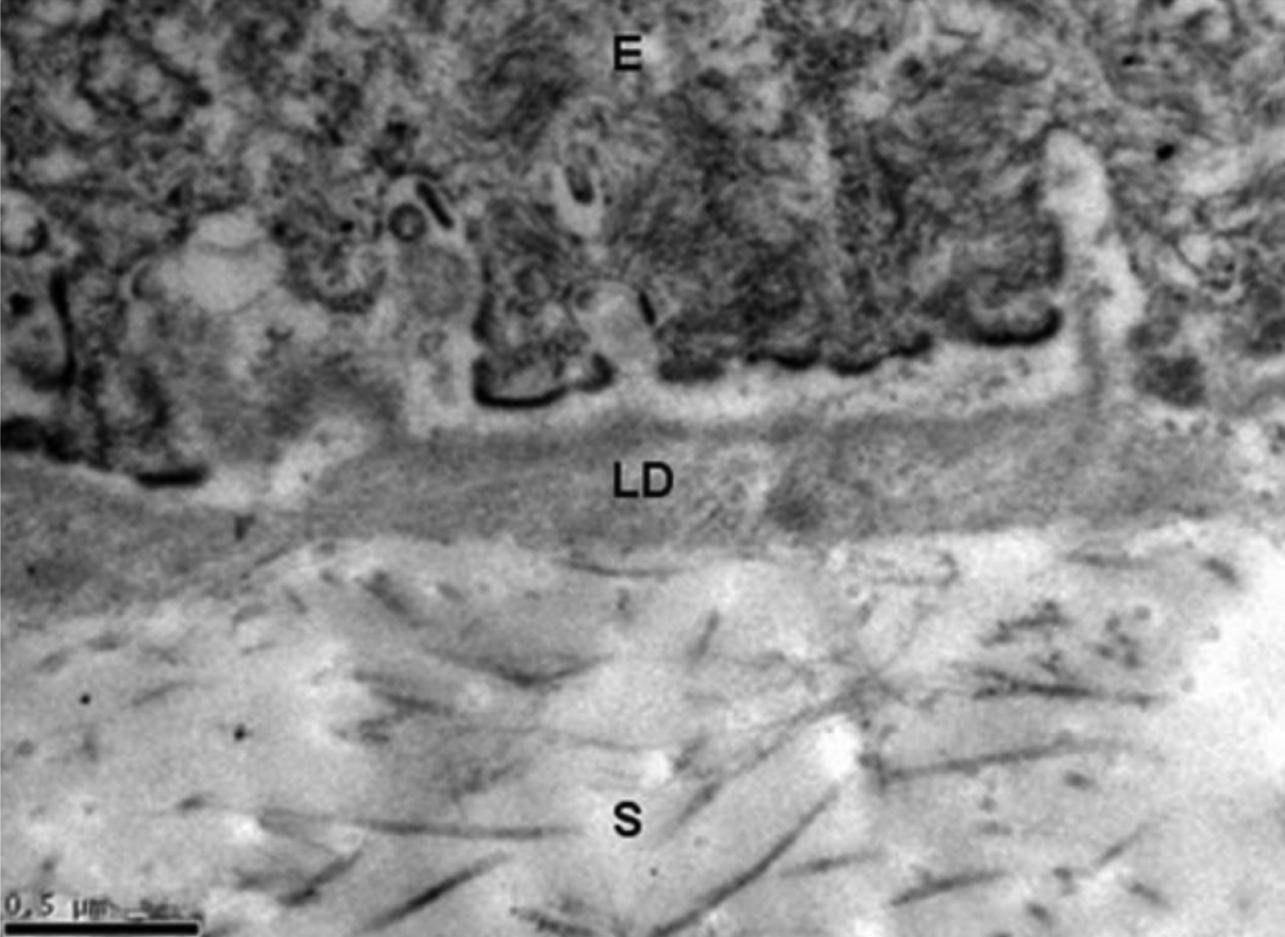
Transmission electron micrograph of untreated HAM tissue (normal control). The basement membrane supports the overlaying epithelium and is composed of a three-layered basal lamina and lamina fibroreticularies. Lamina rara externa (lucida) is an electron-lucent zone directly bordering the adjacent cell which makes up the upper portion of the basal lamina. Lamina densa (LD) is an electron-dense zone that appears somewhat amorphous and granular, and constitutes the intermediate part of the basal lamina. Lamina rara interna comprises the basal portion of the basal lamina. The three layers of basal lamina sit on top of the lamina fibroreticularies, which is synthesized by cells from underlying connective tissue and contains fibrillar structures namely anchoring fibrils, elastic fibrils and microfibril bundles. In the image, “E” indicates epithelium, “LD” indicates lamina densa, and “S” indicates stroma. Magnification: 30, 000×, the scale bar indicates 0.5µm.

A transmission electron micrograph of untreated HAM tissue is shown in Figure 4. The BM supports the overlaying epithelium and is composed of a three-layered basal lamina and lamina fibroreticularies. Lamina rara externa (lucida) is an electron-lucent zone directly bordering the adjacent cell which makes up the upper portion of the basal lamina. Lamina densa (LD) is an electron-dense zone that appears somewhat amorphous and granular, and constitutes the intermediate part of the basal lamina. Lamina rara interna comprises the basal portion of the basal lamina. The three layers of basal lamina sit on top of the lamina fibroreticularies, which is synthesized by cells from underlying connective tissue and contains fibrillar structures namely anchoring fibrils, elastic fibrils, and microfibril bundles.

**Figure 5:**
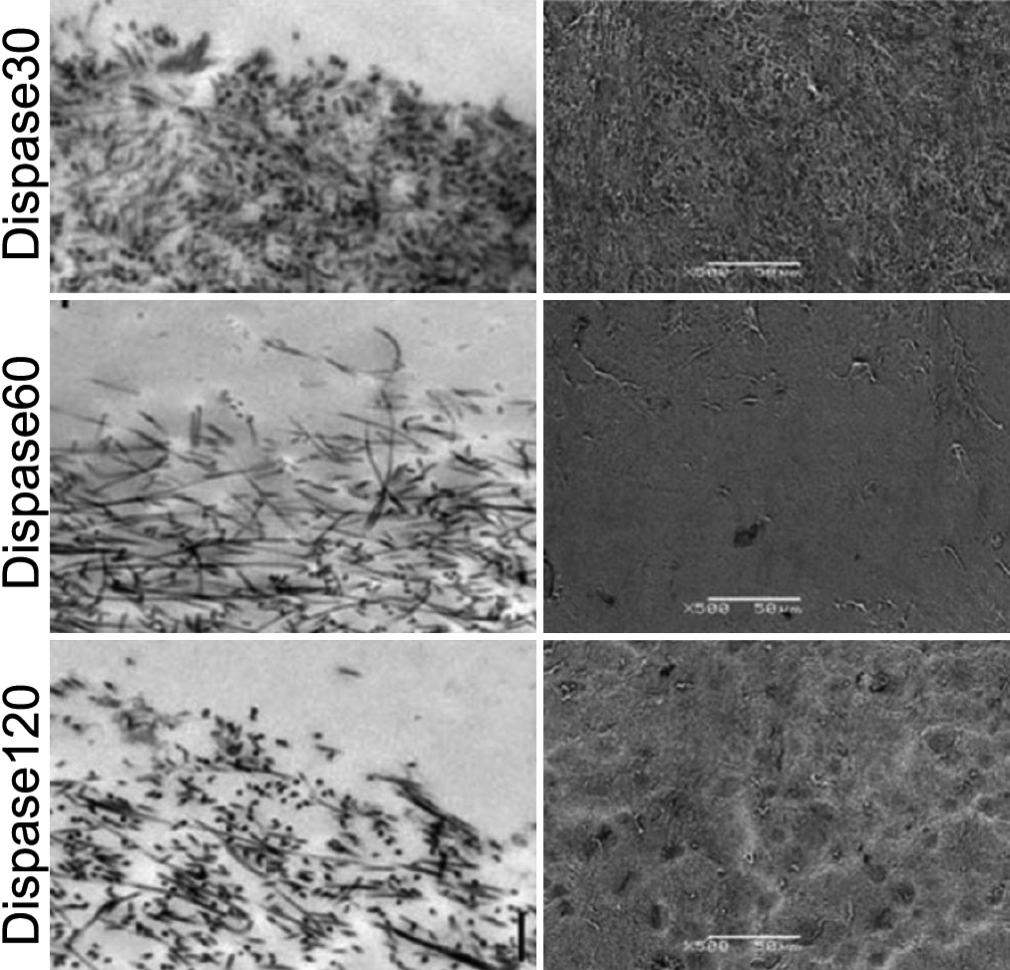
Comparison of the effect of denuded HAM by means of an enzyme, Dispase and mechanical scraping of epithelial cells after incubation. Left panel: Transmission electron micrograph showing the effect of different incubations with Dispase for 30, 60, or 120 min on HAM. After 30 min incubation with Dispase, basal cells of the remnant epithelium sent out cytoplasmic blebs. After 60 min, the lamina densa was disrupted with exposure of stromal collagen. After 120 min, further loosening of the collagen structure occurred. Magnification: 30, 000×, the scale bar indicates 0.5µm. Right panel: Scanning electron micrograph showing the surface of denuded HAM by Dispase. Longer incubations produced a smoother surface with a more open collagen structure.

Transmission electron micrographs showing the effects of different incubations with Dispase for 30, 60, 120 min on HAM are shown in Figure 5 (left panel). Shorter incubations (30 and 60 min) with Dispase caused less disruption of the lamina densa of the epithelial basal lamina. After 30 min incubation with Dispase, basal cells of the remnant epithelium sent out cytoplasmic blebs. After 60 min, the lamina was completely disrupted with no epithelial cell remnants and exposure of the stromal collagen. After 120 min, further loosening of the stromal collagen network was seen.

Evaluation of the quality of the surface of DHAM by scanning electron micrographs showed that longer incubations produced a smoother surface (Figure 5, right panel).

## Discussion

In this study on the effects of Dispase II treatment on HAM, our results indicate that Dispase II treatment is associated with the digestion of several ECM molecules and growth factors, leading to the disruption of HAM ultrastructure and alteration of the surface morphology.

DHAM has been used in ocular surface reconstruction as a substrate for in-vitro corneal, limbal, and conjunctival cell expansion, with several reported advantages over intact HAM sheets. Corneal cells grown on DHAM migrate more quickly, assume a more differentiated phenotype, and have a smoother and more regular structure than those grown on intact HAM [19]. Similarly, limbal cells grown on DHAM are well attached and morphologically superior to those grown on intact HAM [17].

The two main approaches currently used to denude HAM for ocular surface cell cultivation have employed chemical or enzymatic treatments. Chemical reagents that have been used include EDTA [19], urea [29], and ethanol [30], while the main enzymes that have been used are dispase [21,22], a combination of trypsin/EDTA [22], and, more recently, thermolysin [18]. A significant potential limitation of most of these methods is the disruption of the BM induced by chemical or enzymatic treatment. The use of thermolysin, a heat-stable metalloproteinase isolated from *Bacillus srearothermophilus,* has recently been reported to effectively denude BM while causing minimal disruption of the BM [18]. This has however only been reported in one study on HAM to date, with no studies on its efficacy in supporting cell growth.

The use of Dispase II in the processing of DHAM currently suffers from a lack of standardization, with reported incubation durations ranging from 5 min to 2 h [21,27,28,31,32]. This wide variation is attributable to two factors. First, there is a paucity of data on the effect of the duration of incubation on the ultrastructure and biology of DHAM and second, the manual scraping to remove the loosened epithelial cells from HAM adds a significant element of unpredictability to the quality of the prepared tissue.

The minimum duration of incubation with Dispase II required to ensure complete removal of all epithelial cells from HAM is not well established. Hopkinson et al. [18] evaluated the effects of HAM incubation with Dispase II over shorter time points (for 3 min to 45 min) to assess the completeness of epithelial cell removal. They found that with the addition of mechanical scraping, complete epithelial cell removal was achieved after 10 min of incubation. We chose to compare time points of 30 min to 120 min since this is the current time used by several centers for incubation before epithelial denudation [21,27,28]. Our results also demonstrated the ultra-structural changes occurring with current clinical protocols on HAM, i.e. at 120 min [27]. TEM of DHAM specimens in our study showed that following scraping, epithelial cells and basement membrane complex were not present after 30 min of incubation, and after 60 min of incubation there was further disruption of the underlying stromal matrix.

Immunofluorescent staining of DHAM in our study revealed the substrate specificity of Dispase II on HAM. Collagens I and II, which constitute the bulk of the fibrous skeleton of HAM, were not markedly affected by Dispase II incubation. Collagen VI is an unbanded filament that is believed to function as a binding element that maintains the structure of the fibrous component and binds it to the BM. As such, collagen VI expression was unchanged with incubation up to 60 min, but prolonged incubation to 120 min led to a loss of collagen VI expression that corresponded to a loosening of the stromal structure. The greatest changes in the extracellular matrix were seen for proteins associated with the BM. Dispase II has been shown to be relatively specific for collagen IV and fibronectin [23], and expression of these components was absent after 30 min, similar to the findings of Hopkinson et al. [18]. Dispase II is not believed to cleave laminin [23]. Laminin was however undetectable in DHAM in our study and the study by Hopkinson. Laminin is distributed in the lamina lucida of the BM, serving as an anchoring filament to the underlying lamina densa [33], and our immunoflourescence and TEM findings imply that the plane of cleavage of Dispase II has to be subjacent to the lamina densa. Our results also showed an absence of fibronectin expression after Dispase II treatment. This is an interesting finding, as Bhatia et al. [34] have shown that dermal fibroblasts seeded onto a chemically denuded DHAM preparation are able to recognize the small amounts of fibronectin in DHAM and bind via integrin-fibronectin interactions. These fibroblasts subsequently secrete fibronectin and assemble a new ECM on the DHAM, and are also stimulated by DHAM to secrete several key cytokines involved in chemotaxis and wound healing including IL-1 to -7, IFNs, TNF-α, and MIPs. The interaction between fibronectin and fibroblasts may be a key mediator of cell growth and wound healing augmentation by DHAM may be adversely affected by Dispase II digestion.

Several growth factors have been investigated for their roles in promoting epithelialization on HAM. High levels of EGF, KGF, HGF, TGF-β1, FGF, and several other growth factors [9,35] have been identified in HAM, most of which, particularly EGF, TGF-β1, and FGF, have well documented roles in the epithelial-mesenchymal cell interactions involved in corneal wound healing [36]. As far as we are aware, our study is the first to asses the effects of Dispase II incubation on growth factor expression in DHAM. The levels of these growth factors in DHAM, with the exception of FGF-2, PDGF-B and KGF, were reduced by Dispase II incubation. Our findings are in agreement with the general belief that Dispase II is not a broad-spectrum protease [23].

In conclusion, the use of Dispase II in the preparation of DHAM causes significant changes to the ultrastructure of the membrane, particularly the BM. Although our study did not assess the viability of DHAM as a substrate for cell growth, Ang et al. [27] have reported that conjunctival epithelium can be successfully grown on DHAM incubated with Dispase II for 2 h. It has been shown that limbal epithelial explants grown on HAM disassemble the BM prior to secreting a new BM [37]. This suggests that an intact BM is perhaps not of critical importance in epithelial cell expansion on HAM. However, we have noted from our experience that cell outgrowth from explant culture is not consistently achieved every time on dispase treated HAM. Our results indicate that the prolonged incubation with dispase may cause significant disruption in HAM structure and affect cell growth in some cases. Further work to characterize limbal, corneal, and conjunctival cell growth on DHAM is warranted.
